# Jumping Performance and Behavior of the Globular Springtail *Dicyrtomina minuta*

**DOI:** 10.1093/iob/obae029

**Published:** 2024-08-29

**Authors:** A A Smith, J S Harrison

**Affiliations:** Biological Sciences, North Carolina State University, Raleigh, NC 27695, USA; Research and Collections, North Carolina Museum of Natural Sciences, Raleigh, NC 27601, USA; School of Chemical and Biomolecular Engineering, Georgia Institute of Technology, Atlanta, GA 30318, USA

## Abstract

Springtails are among the most abundant arthropods on earth and they exhibit unique latch-mediated spring-actuated jumping behaviors and anatomical systems. Despite this, springtail jumps have not been well described, especially for those with a globular body plan. Here, we provide a complete description and visualization of jumping in the globular springtail *Dicyrtomina minuta*. A furca-powered jump results in an average take-off velocity of 1 ms^−1^ in 1.7 ms, with a fastest acceleration to liftoff of 1938 ms^−2^. All jumps involve rapid backwards body rotation throughout, rotating on average at 282.2 Hz with a peak rate of 368.7 Hz. Despite body lengths of 1–2 mm, jumping resulted in a backwards trajectory traveling up to 102 mm in horizontal distance and 62 mm in vertical. Escape jumps in response to posterior stimulation did not elicit forward-facing jumps, suggesting that *D. minuta* is incapable of directing a jump off a flat surface within the 90° heading directly in front of them. Finally, two landing strategies were observed: collophore-anchoring, which allows for an immediate arrest and recovery, and uncontrolled landings where the springtail chaotically tumbles. In comparison to other fast jumping arthropods, linear performance measures globular springtail jumps place them between other systems like fleas and froghoppers. However, in angular body rotation, globular springtails like *D. minuta* surpass all other animal systems. Given the extraordinary performance measures, unique behavioral responses, and understudied nature of these species, globular springtails present promising opportunities for further description and comparison.

## Introduction

For arthropods, jumping can be used as a means of locomotion, a start to a wing-powered flight, or a rapid escape from a predator. Arthropods that perform the fastest jumps rapidly release stored elastic energy, usually to spring away from danger (Sutton et al. 2019). These jumping apparatuses are referred to as latch mediated spring-actuation systems, or LaMSA systems (Longo et al. 2019). These LaMSA jumpers exhibit some of the fastest recorded animal accelerations (Burrows 2014), and include well-studied organisms such as grasshoppers (Bennet-Clark 1975), fleas (Sutton and Burrows 2011), and plant-feeding hemipterans (e.g., Burrows 2006; 2014). Collembolans, or springtails, (Subphylum: Hexapoda; Class: Collembola) are known for their impressive spring-driven jump, which they use primarily as an escape jump from predators. Springtails are one of the most abundant arthropods on earth (Potapov et al. 2023), with over 8000 described species found on a variety of substrates, including decaying wood, water surfaces, vegetation and, most commonly, amongst the soil and leaf litter (Hopkin 1997). Despite their broad diversity and abundance, springtail jumps have not been well explored or described. Furthermore, springtails exhibit behaviors and anatomical LaMSA features not found in other arthropod systems.

The majority of described arthropod LaMSA jumping systems represent derived functions of body parts (or whole bodies) that also move and are used in other non-LaMSA modes. For example, a froghopper uses its hind legs to walk and for a LaMSA jump (Burrows 2006), a trap-jaw ant uses its mandibles to manipulate soil and larval nestmates as well as perform a rapid snap (Patek et al. 2006), and a gall midge fly larva still undulates its body to move but also loops its body for a spring-actuated jump (Farley et al. 2019). The collembolan furca, however, the part of a LaMSA apparatus used to propel the springtail off the substrate, has no apparent use other than rapidly powering a jump. The furca (also called the furcula) is a modification of the fourth abdominal segment and is held, folded underneath and against the body (Oliviera and Smith 2024). Whether living on wood, water, or soil, the furca is used for rapid escape jumps. Though some species, usually those restricted to specific environments, have lost a functional furca, for instance, cave dwelling (Pomorski et al. 2009) and intertidal zone (Davenport 1903) species.

The morphology of the furca and the jumping apparatus have been studied in detail (Manton 1972; Eisenbeis and Ulmer 1978; Christian 1979; Brackenbury and Hunt 1993; Sudo et al. 2013b). Recent work incorporates µCT morphological reconstruction techniques to describe abdominal musculature and its integration with abdominal basal plates and rods that are likely candidates for energy-storing springs used to power the furca flicks (Oliveira 2022; Oliveira and Smith 2024), as well as structures that likely serve as a latching/release mechanism (Rillich and Oliveira 2023). Detailed descriptions of how this jumping apparatus works based on high-speed photography are not as numerous, likely because of technical challenges presented by their small size and extreme speeds. Early insights and visualizations of collembolan jumping mechanics are from Christian (1979), who filmed jumps of both segmented (Order: Poduromorpha and Entomobryomorpha) and globular (Order: Symphypleona) springtails at frame rates between 750 and 1600 frames per second. Though many aspects of the jumps could not be resolved at those frame rates and with silhouette images, this early work reported jumping speeds exceeding 1 m/s and liftoff durations of less than 1 ms. Later anecdotal descriptions of a semi-aquatic segmented springtail jump, filmed at 20,000 frames per second, report similar jump performance measures (Sudo et al. 2015). To date, the most thorough description of springtail jumping is Ortega-Jimenez et al.'s (2022) description of the segmented springtail *Isotomurus retardatus*. They document this semi-aquatic springtail's ability to jump off water and travel up to 48 body lengths in distance, reaching just over 0.7 m/s in speed. Beyond initial take-off performance measures, they also describe behavioral aspects of the full jump and landing, such as the springtail's use of the collophore, or ventral tube, to gather a water droplet at take-off in order to orient its ventral surface down and adhere to the water surface upon landing. Our clearest images and deepest understandings of the springtail jump are from these studies of segmented, semi-aquatic springtails. However, terrestrial springtails, especially those with a globular body plan, likely exhibit very different jumping strategies.

Thorough kinematic descriptions of globular springtail jumps have yet to be made. Two published accounts provide some jump performance measures of globular species. One of the first detailed descriptions of springtail jumps by Christian (1979) includes an account of one globular species, *Sminthurus viridis*. Data presented in this study are from recordings captured at 1250 frames per second. At this temporal resolution, the take-off sequence is limited to only a single frame that captures the springtail as it pushes off the ground. A more recent description of globular springtail jump kinematics was done by Sudo et al. (2013) with *Bourletiella hortensis*. This study presents a more finely resolved picture by describing jumps captured at frame rates between 4500 and 27,000 frames per second. However, the data presented in the study are anecdotal descriptions of single jumps filmed at different perspectives instead of descriptions based on replicated data sets. The measures that these studies present describe globular springtail jumps as extraordinary as compared to segmented springtails. Reported takeoff accelerations in globular springtail species range from 970 to 1800 m/s^2^, which outperforms the segmented springtail species with maximum reported accelerations are between 600 and 800 m/s^2^ (Christian 1979; Sudo et al. 2015). However, in addition to linear movement, globular springtail jumps involve rapid backward rotation while taking off. The reported rotational rates of 472 (Christian 1979) and 417 hz (Sudo et al. 2013) exceed those of all other arthropods that rapidly rotate their bodies during jumps, such as whiteflies (58.3 hz: Ribak et al. 2016), trap-jaw ants (67 Hz: Patek et al. 2006), flea beetles (187 Hz: Brackenbury and Wang 1995), pygmy mole crickets (190Hz: Burrows and Picker 2010), and jumping plant lice (336 Hz: Burrows 2012). Some of these insects (e.g., plant lice: Burrows 2012; and flea beetles: Zong et al. 2023) can dampen and stop body rotation in air by deploying their wings. Whereas others, globular springtails included, rotate throughout the entirety of their jump trajectory and seemingly ending in a chaotic and unpredictable bounce and roll.

In this study, our goal is to provide a complete description and visualization of jumping in the globular springtail species *Dicyrtomina minuta* (Fabricius, 1783). A recent previous study described the anatomy of the *Dicyrtomina* jumping apparatus and performance measures relative to the release and extension of the furca (Oliviera and Smith 2024). Here, we visualize take-off, trajectory, and landing and provide relative performance measures. In addition, we describe behaviors related to jump distance and direction in response to different stimuli and behaviors involved in controlling the landing process.

## Methods

### Study species

**Fig. 1:**
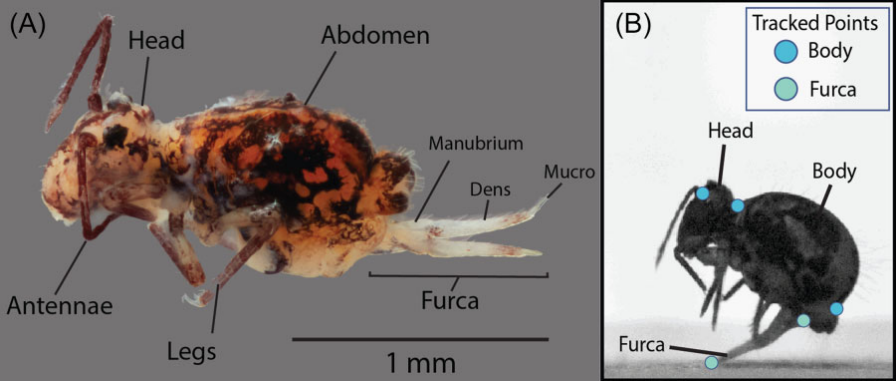
*Dicytromina minuta* springtails are found in the top layers of leaf litter. (A) Light microscope image of *D. minuta* showing a lateral view with posterior end of the animal to the right. Anatomical reference points relevant to body orientation and jumping morphology are shown on the panel. The collophore is underneath the body and not everted and therefore not visible in the image. (B) A *D. minuta* springtail during a jump where the furca is pushing down on the substrate and lifting the springtail into a jump. Circles on the body represent the points tracked during a jump and used to calculate jump kinematics.

Study specimens of *D. minuta* (Fig. 1) were collected live sifting leaf litter through a metal mesh screen in the author's (AAS) residential neighborhood in Raleigh, NC, USA, between the months of January and March, in the years 2020–2023. Identification of species was provided by Dr. Aron Katz (University of Illinois, Urbana-Champaign) based on physical samples.

### Take-off

To describe jump take-off, we collected 12 close-up and high-speed sequences from different individuals, one jump per individual. The springtails jumped off a flat piece of acrylic (6 cm × 1.9 cm) covered in a general-purpose beige masking tape (Duck^®^ brand). The tape provided a uniform textured material to increase friction but maintain a flat substrate. An LED array (Neewer^®^ CN-160) behind the platform aimed directly at the lens through Rosco Roscolux #116 Tough White Diffusion filter paper provided a uniform white background. The springtail and platform were illuminated by a high-intensity 12,000-lumen LED array (Visual Instrumentation Corporation). The springtails were given no stimulus to encourage their jump other than light. The recorded images had a horizontal field of view of approximately between 7.6 and 5.8 mm and a vertical view above the platform of approximately 3.8–5.25 mm. The pixel resolution of the recordings was 640 × 480, captured at 40,322 frames per second with a 24.8 µs exposure, using a Phantom VEO 1310 camera with a Canon MPE-65mm f/2.8 1–5x Ultra-Macro with the aperture set at f/8. Immediately after capturing a set of takeoff jumps, a ruler was placed in the focal plane of the camera and recorded to calibrate scale.

Using dltdv8 software in MATLAB (DLTdv8 MATLABscript; MATLAB9.4, version R2018a; Hedrick 2008) we tracked springtail movement starting just starting five frames before furca release and ending after the springtail reached ballistic motion. Points on the body included the base of the antennae, the connection between the head and the fused thorax, and the posterior tip of the abdomen (Fig. 1). We also tracked points at the tip and base of the furca (Fig. 1) to quantify the rotation of the furca during take-off. From tracked points on the body, we averaged the X and Y values to get a rough approximation of the center of the mass and then used the approximated centroid to calculate body dynamics. Body angle relative to the ground was measured using the rotation of the points at the base of the antennae and the posterior tip of the abdomen relative to the angle of the substrate in the frame. The rotation of the furca was measured using four points (the base of the head, the posterior point of the abdomen, the base of the furca, and the tip of the furca). Velocity and acceleration were measured using derivatives of a fifth-order polynomial model fit to the raw displacement data with respect to time. One random jump was digitized 5 times to calculate the digitizing error. The standard error of the reported mean from digitizing constituted 0.4% of the reported body take-off velocity measurement, 1.5% of the reported body acceleration measurement, 0.5% of the reported body angular velocity measurement, and 0.8% of the reported furca velocity measurement. All kinematics were analyzed and visualized with custom-written code in R (v 2023.03.1) and are available in supplementary materials.

For a measurement of total body mass, we averaged individual live weights of 20 springtails. The 20 individuals weighed were the same as those used in the “Distance and direction in response to a directed stimulus” section below. We used a Mettler Toledo WXTS microbalance with 0.001 mg readability. The average live body weight was 0.743 mg with a range of 0.231–0.983 mg.

To determine whether the jump of *D. minuta* is spring-driven, we calculated the mass-specific maximum power output of the jump and compared it to the previously measured maximum power outputs of vertebrate muscle (1200 W kg^−1^; Askew et al. 2001). To calculate the mass-specific power output of the jump, we first calculated the jump energy (E).


\begin{equation*}
E = 0.5\ {\mathrm{m}}{{{\mathrm{v}}}^2}.
\end{equation*}

Where v is the peak velocity of the springtail at take-off and m is the mass of the springtail. Unfortunately, we were unable to directly measure the mass of the springtails in the take-off kinematic dataset. Instead, we used the average, minimum, and maximum mass measured from separate individuals as approximates in our energy calculations. We then divided the jump energy by the duration of the jump to determine the jump power (P), where *d* is the duration of the jump in seconds.


\begin{equation*}P = \frac{E}{d}.\end{equation*}

Finally, the mass-specific power output, also known as power density (PD), was calculated by dividing the power by the muscle mass (M_musc_).


\begin{equation*}
PD = \frac{P}{{{{M}_{{\mathrm{musc}}}}}}.
\end{equation*}

Currently, the precise muscles responsible for generating the energy for the jump in springtails are still unknown. To approximate PD, we used micro-CT data from *Dicyrtomina ornata* (Oliviera and Smith 2024), which revealed that the volume of the muscles associated with the furca of the fourth abdominal segment is about 7.15% of the total body volume. These muscles include both the flexor and extensor muscles that actuate the furca. Therefore, for our first approximation, we used 7.15% of the body mass as M_musc_. However, as a more conservative approximation, we also calculated the energy density after assuming that M_musc_ was 25% of the total body mass. Both methods are overestimates for M_musc_ and would underestimate the PD. Comparing these estimations to the maximum potential power outputs of muscle allows for a clear determination of whether the movement could be powered by muscle or whether it requires an elastic energy storage system.

### Full jump trajectory

To describe aerial behavior, we filmed individuals (*n* = 15) jumping off an acrylic platform (0.5 cm tall, as described above) set on a larger grided platform. This allowed the camera to be positioned nearly perpendicular to the plane of the platform. In the recordings, the horizontal field of view ranged from 8.57 to 9.02 cm with a vertical view of 5.8–6.4 cm above the platform. The pixel resolution of the recordings was 896 × 720, captured at 4300 frames per second with a 232.5 µs exposure, using a Phantom Miro LC321S camera with a Venus Optics Laowa 60 mm f/2.8 2x Ultra-Macro with the aperture set at f/11. The scene was illuminated from behind with a diffused light and above from a high-powered LED array as described above. The springtails were given no stimulus to encourage their jump except for the light.

We tracked the centroid of the body every five frames starting from the first noticeable upward movement of the body. We ceased tracking when the springtail first contacted the platform or broke the would-be plane of the platform when they landed beyond the jumping surface. For rotational data, we noted the frame at which one body rotation was completed throughout the entire jump duration. We also noted the frame in which the furcula was folded back underneath the body. Tracking of body centroid, rotations, and refolding of furca was done manually, frame-by-frame in ImageJ. The resulting data were analyzed and visualized using custom-written code in R.

### Distance and direction in response to stimuli

We filmed jump sequences from above to compare the direction and distance of a jump in response to a universal stimulus (bright light) and a directed stimulus (bright light + contact with a paintbrush). The data set in response to a universal stimulus were the same 15 jumps analyzed and outlined in the “Trajectory and performance” section above. However, instead of analyzing the profile view of the jumps, we used the recording from a second camera (Nikon D5300) mounted directly above the platform, recording at 60 frames per second, with a pixel resolution of 1920 × 1080 and field of view of 97 cm × 55 cm, through a Sigma 28–100 mm zoom lens set to 35 mm. From these recordings, we used the frame directly preceding the jump and the frame in which first contact is made with either the acrylic jumping platform or the broader surface of the copy stand 0.5 cm below it. The distance was determined using a 5-cm grid printed on the surface of the copy stand and the direction was relative to the springtail's original orientation. The universal stimulus to induce jumping was a high-intensity 12,000-lumen LED array (Visual Instrumentation Corporation) mounted above and slightly (∼25°) in front of the jumping platform. The beam from the light illuminated both the jumping platform and the surface below in entirety.

A second set (*n* = 20 individuals) of jump sequences was filmed but with an additional directed stimulus. The same lighting conditions were used as in the set immediately above however the jumps were in response to being touched at posterior end with the bristles of a fine paintbrush, a method used by others to assess escape-jumping behavior in segmented springtails (Bauer and Christian 1987). All recorded jumps were performed directly after physical contact with the brush. In this set, instead of jumping off the acrylic platform, the springtails were placed directly onto the hard plastic surface of the copy stand, which was covered in a layer of general-purpose beige masking tape (Duck^®^ brand) to provide a more textured but still flat surface identical to that of the smaller acrylic platform. The jumps were captured by a Phantom Miro LC321S camera recording at 1000 frames per second, with a pixel resolution of 1920 × 1080 and a field of view of 115 cm × 65 cm through a Sigma 28–100 mm zoom lens set to 35 mm. As above, distance and direction were determined using the frames at the start of a jump and first ground contact, using a 5-cm grid printed on the surface of the copy stand and relative to the original direction the springtail was facing.

### Landing

We used two video data sets to describe how *D. minuta* interacts with the ground during landing. First, we filmed a set of close-up videos filmed laterally to visualize how the springtail interacts with the ground during landing. To capture this, the high-speed camera was positioned perpendicular to a flat piece of acrylic (60 mm × 39 mm) covered in a general-purpose beige masking tape (Duck^®^ brand). The recorded images had a horizontal field of view of approximately 37 mm and a vertical view above the platform of approximately 18 mm. The pixel resolution of the recording was 1280 × 720, captured at 1500 and 40,000 frames per second with a 333 and 250 µs exposure, using a Phantom VEO 1310 camera with a Venus Optics Laowa 60 mm f/2.8 2x Ultra-Macro with the aperture set at f/11. The camera position and focus were static, and the springtail jumps were stimulated by touching them with a paintbrush while they were freely walking behind the platform. Recordings were only saved if the springtail landed in-frame and within the focal plane of the camera, which was only achievable by chance. We captured 14 of these sequences, each with a different individual. From these recordings, we could observe body orientation at landing, whether and how the collophore was used, and the time from touch down to standing.

Additionally, we used the video dataset collected for measuring jump direction to expand our dataset for measuring self-righting duration. From these recordings, we assessed the righting time, distance traveled, and whether the collophore was used to stop the springtail during the landing process. Collophore involvement was evident through observing an interrupted bouncing trajectory after landings as well as the springtail rebounding when the collophore remained attached to the landing substrate.

## Results

### Liftoff

Upon release of the furca, the manubrium and dens rapidly rotated away from the body until the tips of the dens arms, the mucro, made contact with the substrate. The duration between initial release and contact with the substrate occurred between 0.07 and 0.17 ms. Upon making contact, the furca would deform, bending slightly at the dens-manubrium joint. As the energy was transferred into the substrate, the body would begin lifting, starting with the anterior portion first, rotating the head backward. The body rotation before take-off was around 69.4 deg (SD = 15.8 deg, *N* = 12). As the body lifted off and the furca continued to push off the substrate, the slight flexion in the furca would recoil returning to its original shape, as further described in Oliveria and Smith (2024).

**Fig. 2:**
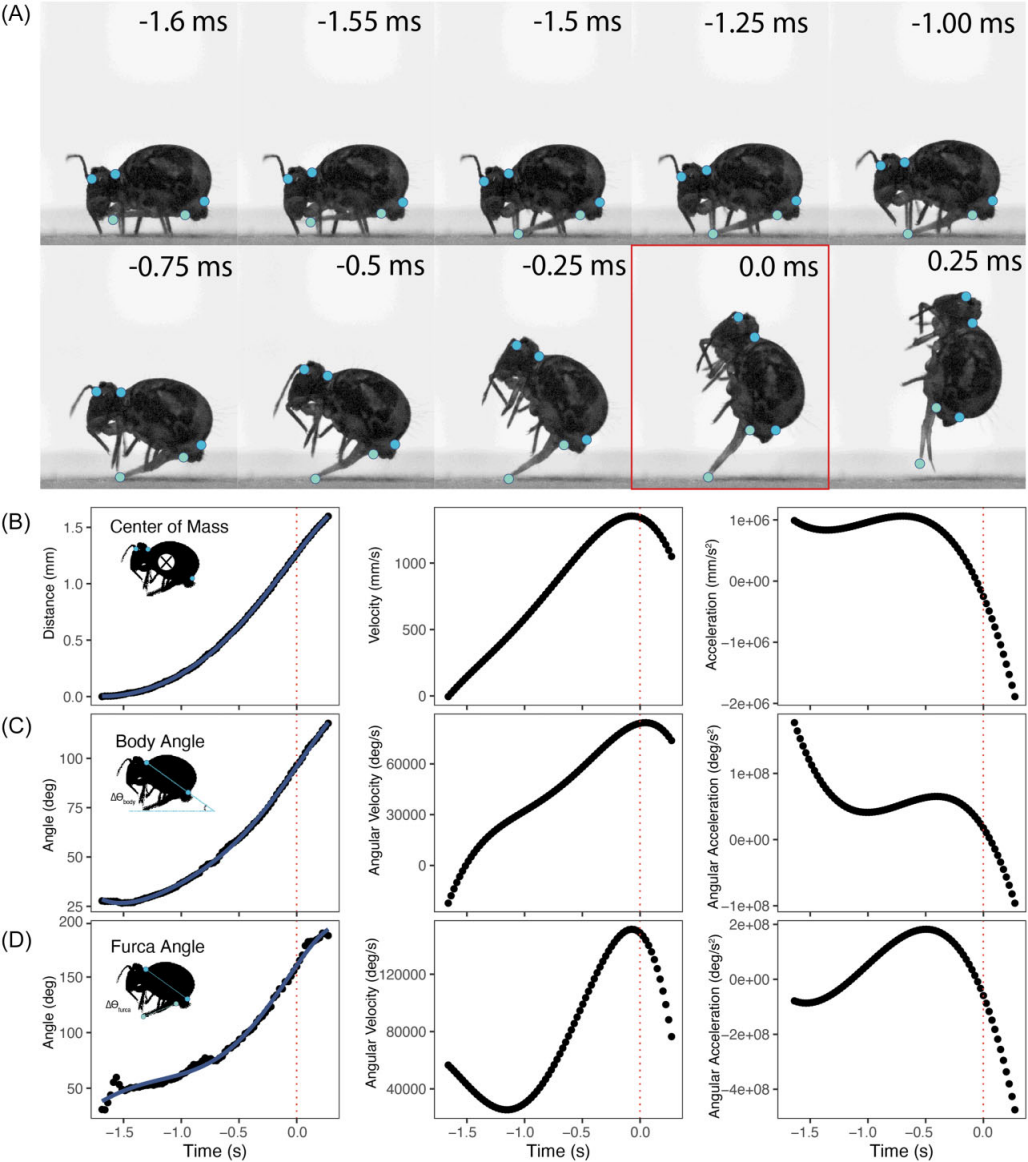
Liftoff process from furca release until airborne. (A) Anatomical reference points and sequential extracted frames from a single sequence captured at 40,322 frames per second showing furca release, body rotation, and initial liftoff. Time stamps indicate milliseconds relative to the last point of contact with the ground (outlined). (B) Distance, velocity, and acceleration measured from centroid point of the body during the sequence shown in A. Dotted line indicates last point of ground contact. (C and D) Body and furca angle relative to the ground, rotational velocity, rotational acceleration during the sequence shown in A. Dotted line indicates last point of ground contact.

Take-off duration was on average 1.7 ms (SD = 0.5 ms, *N* = 12). Average takeoff speed was as high as 1.52 m/s with an average of 0.98 m/s (SD: 0.3 m/s, *N* = 12). The furca opened at an average rate of 3.1 × 10^5^ deg/s pushing off the ground and reaching an average change of 135.4 degrees before the springtail reached ballistic motion. The average take-off angle for jumps was 109.5 degrees, which suggests jumps would be directed backward. An example set of measurements from a single jump are illustrated in Fig. 2 (also see supplementary video), summary statistics for all jumps can be found in Table 1.

**Table 1:** Jump kinematics, mean (min–max; sample size), of *D. minuta* and other globular springtails

Variable	*Dicyrtomina minuta*	*Bourletiella hortensis* ^1^	*Sminthurus viridis* ^2^
Body mass (mg)	0.74 (0.23–0.98; 20)	1.3	0.335
Body length (mm)	1.53 (1.29–1.86; 12)	–	–
Furca length	0.83 (0.73–1.07; 12)	–	–
Take-off duration (ms)	1.7 (1.1–2.9; 12)	1.12	0.8
Peak take-off velocity (ms^−1^)	0.982 (0.436.4–1.524; 12)	1.98	1.39
Peak take-off acceleration (ms^−2^)	1,582.3 (511.6–1,938.8; 12)	1800	970
Peak body angular velocity (rads^−1^)	1,773.0 (1,141.0–2,316.4; 12)	2620	2967
Furca displacement (to take-off) (deg)	135.4 (94.4–163.1; 12)	–	–
Peak furca angular velocity (rads^−1^)	5,478.5 (2,499.4–7,844.6; 12)	–	–
Jump height (mm)	32.89 (16.08–62.01; 15)	115	97
Jump horizontal distance (mm)	32.6 (6.81–55.04; 15)	–	–
Furcula reset time (s)	0.049 (0.037–0.62; 15)	–	–

1 Data from Sudo et al. 2013.

2 Data from Christian 1979.

When assuming 7.15% of body mass, the approximated mass-specific power of *D. minuta* jumps was on average 5167.3 W kg^−1^ and ranged from 462.9 to 13941.4 W kg^−1^ (Supplemental Fig. S1). When assuming the muscle mass was 25% of the body mass, the approximated mass-specific power of *D. minuta* jumps was on average 1477.83 W kg^−1^ (Supplemental Fig. S1). Even using both conservative estimates of muscle mass, most measurements exceed the maximum measured power output recorded for vertebrate muscle (1200 W kg^−1^; Askew et al. 2001).

### Trajectory and performance

**Fig. 3:**
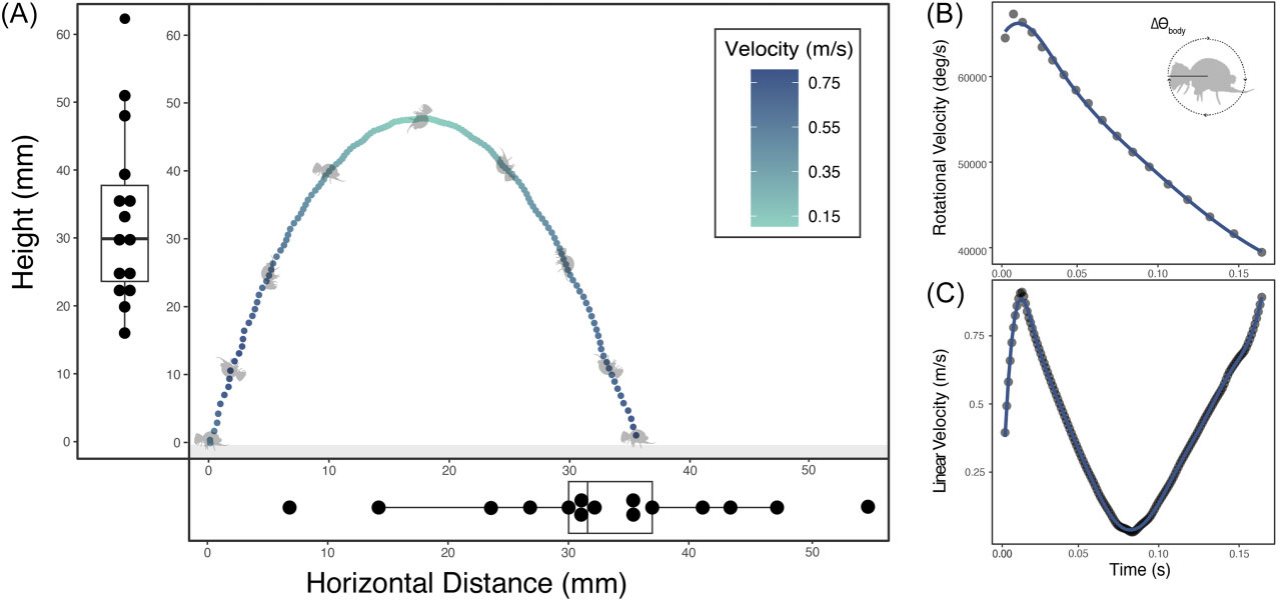
Mid-air trajectory and performance of full jumps. (A) An example trajectory tracking height and horizontal distance of a jump. Darkness value of the point represents velocity as indicated in key. Summary maximum height and distance data for all jumps presented as median, 25% 75%, and range, individuals denoted as points. (B) Rotational velocity measured by the frame at which a flip was completed during the same jump that height and distance was tracked and displayed in panel A. Each point in panel B is complete flip tracked across the mid-air duration of a jump. (C) Linear velocity throughout the complete trajectory of the jump.

**Fig. 4:**
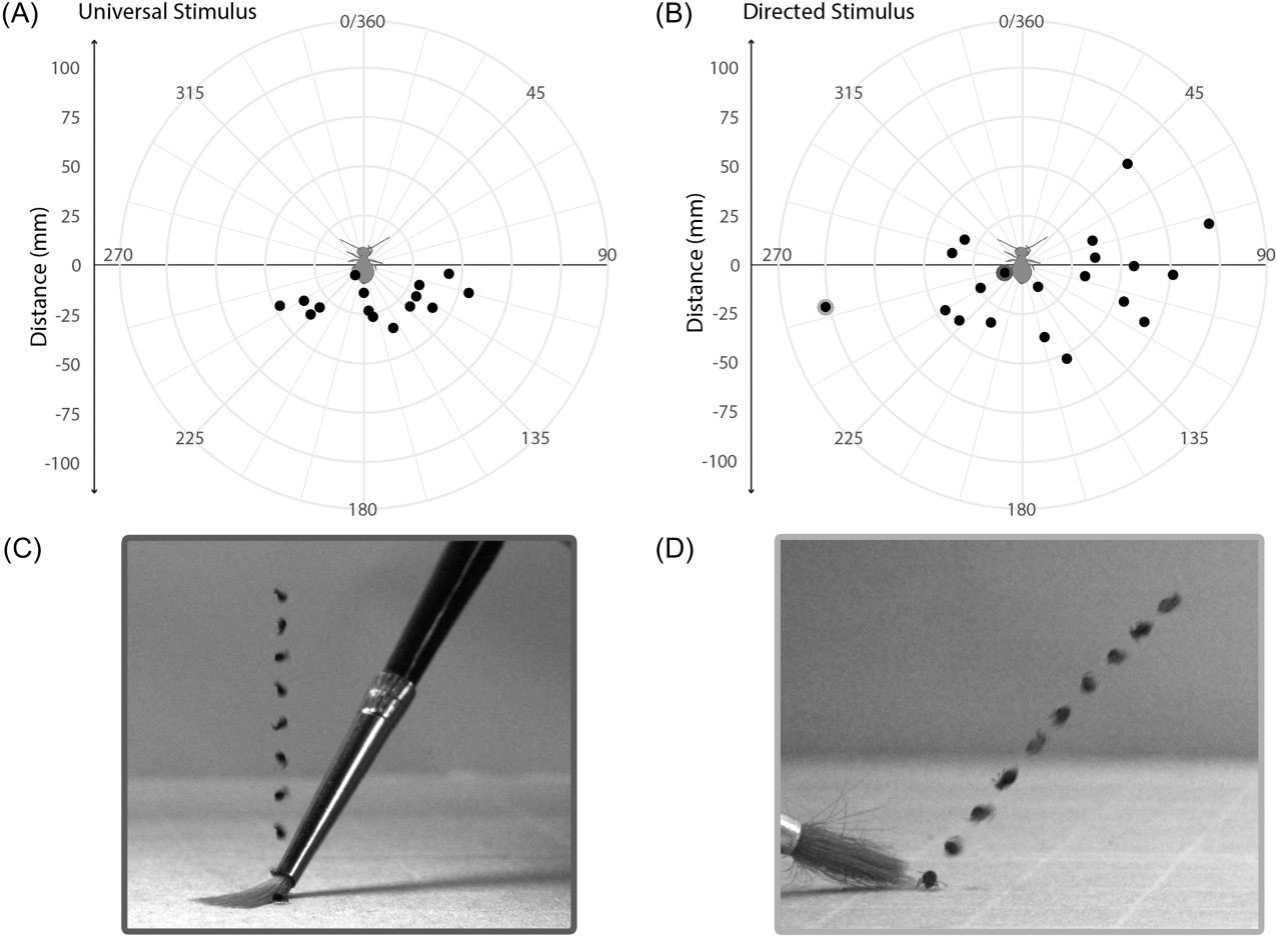
Polar plots displaying jump distance and trajectory in response to a universal stimulus of bright light (A) and a directed stimulus of light plus posterior contact from a paint brush (B). Plots simulate a view from above where the springtail is standing with its head pointed up (forward) and abdomen pointed down (backward). Individual directed stimulus jumps outlined in light and dark values in panel B are depicted in panels C and D. Panel C shows the initial trajectory of the jump with the smallest horizontal distance, while panel D shows the initial trajectory of the jump with the farthest horizontal trajectory.

Vertical height achieved during a jump averaged 32.89 mm (16.08–62 mm; Fig. 3A), while horizontal distance averaged 32.6 mm (6.8–55.04 mm; Figs. 3A, 4A). Furcula retraction most often occurred during the assent phase, on average the furcula was fully retracted within 0.049 s (0.037–0.062 s), which is equivalent to about 31% of the total jump duration (range: 23–42%).

When the springtails reach ballistic motion, their bodies are flipping backward, reaching an average a peak rotational velocity of 101,588 deg/s or 282 Hz (Table 1). The fastest observed rotational velocity measured was 132,717 deg/s or 368 Hz (Table 1). In all jumps, the springtails continue to rotate throughout the entire jump, though peak angular velocity occurred right after take-off and diminished throughout the remainder of the jump. The total number of complete backward body rotations per jump averaged 20.53 (14–29 rotations). From the time of first noticeable movement to start takeoff to the time, the springtail first contacts the ground on its descent, the total time of a jump averaged 161 ms (117.9–231.9 ms). Across their jumps, their average rotational rate was 45,990 deg/s or 127.75 Hz (35,354–58,849 deg/s).

### Distance and direction in response to stimuli

With a universal stimulus (light), jumps resulted in the springtails landing either behind or to either side of where they started, never forward (Fig. 4A; mean direction relative to forward: 168°, min: 96°, max 244°). When jumps were in response to both light and direct posterior stimulation (a touch from a paintbrush) only 6 of the 20 springtails achieved positive forward movement, the majority of jumps saw the springtails land either behind or to the side of where they started (Fig. 4B; mean direction relative to forward: 162°, min: 46°, max 294°). In all observed jumps, the springtails never jumped within a 90° heading directly in front of them. Jump direction was not statistically different between universal and directed stimulus jumps (Wilcoxon rank-sum test, *Z* = 0.417, *P* = 0.68).

Though direction was not different in response to a directed stimulus, horizontal jump distance was. The horizontal jump distance in response to the directed stimulus averaged 48.1 mm (min: 9.9 mm). The maximum observed horizontal distance of 102.1 mm was nearly double that of universal stimulus jumps (Fig. 3A, max: 55 mm). Compared to the horizontal distance of universal stimulus jumps (Fig. 3A), jump distance in response to the directed stimulus was statistically different (Wilcoxon rank-sum test, *Z* = −2.15, *P* = 0.03). Directed stimulus jumps were filmed from an additional side view, however, because the springtails were unrestrained their jumps were often not parallel to the camera's view (Fig 4C–D). Therefore, body angle and trajectory at liftoff could not be reliably measured. However, in general, jumps with longer horizontal distances involved the springtails unevenly leaning their bodies to the side when releasing their furca so that their trajectories were more horizontal (Fig. 4D) as compared to near vertical jumps which resulted in very little horizontal displacement (Fig. 4C).

### Landing

**Fig. 5:**
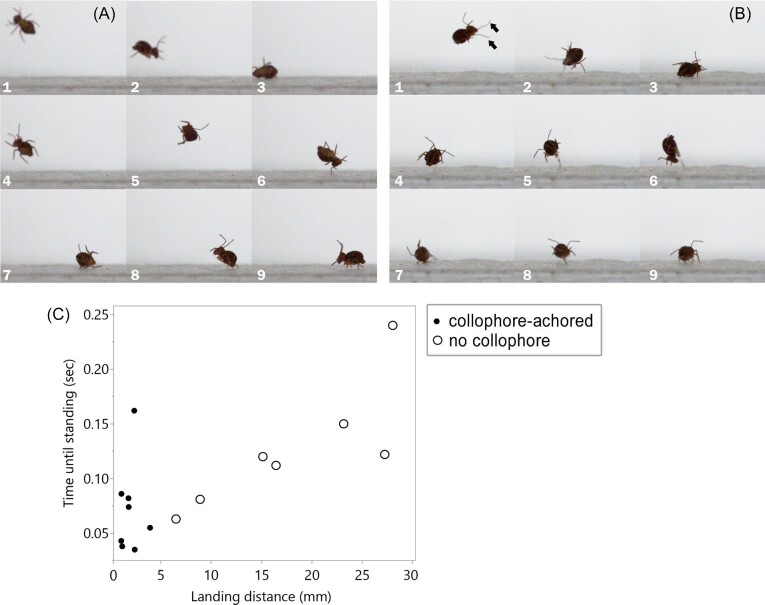
Landing to self-righting process after direct-stimulation escape jumps. (A and B) Close-up high-speed video captures demonstrating two observed landing styles: (A) uncontrolled bouncing (4/14 sequences), (B) collophore anchoring (10/14 sequences). Arrows in panel 1 of C show the branched arms of the collophore extended as the springtail is descending. (C) Time from first touching the ground until first standing compared to horizontal distance traveled during that time. These data are the 15 of the 20 jumps recorded directional escape jumping displayed in Fig. 3B. The remaining five of that data set came to a stop stranded on their backs or did not self-right in the recording.

Through our high-speed video captures with a close-up, perpendicular view of the landing area, we observed *D. minuta* using two strategies during the landing and self-righting process (Fig. 5A–B). One strategy was an uncontrolled series of bounces that diminish to a stop (Fig. 5A). These accounted for 4 of the 14 sequences captured. Of these, initial contact with ground was both dorsal and ventral contact of the abdomen and thorax. Final arrest was observed both in a standing posture and stranded dorsally with legs in the air. Only two of these four sequences captured coming to a rest in a standing posture. The time periods for those two sequences, from first ground contact to standing, were 0.11 and 0.2 s.

A second landing strategy we are calling “collophore anchoring” was more commonly observed (10/14 sequences, Fig. 5B). In these, the collophore is fully everted upon the initial descent and attaches to the ground, dampening the rebound from ground contact. Of these 10 sequences, initial ground contact was both dorsal and ventral contact of the abdomen and thorax, with some resulting in a bounce before the collophore touches the ground. In 6/10 sequences, the collophore attaches to the ground but the bounce of the springtail results in the collophore detaching from the ground. In 9/10 of these sequences, the springtail remains in frame and the collophore is used in the final arrest and a return to standing posture. Average time from initial contact to standing for these nine sequences was 0.108 s (min: 0.052 s, max: 0.205 s).

Through analyzing footage from above were able to record both landing distance traveled and time to self-right after first contacting the ground. Of the 20 sequences in this set of videos, 5 individuals did not self-right within the duration of the recording and were not analyzed. Initial contact with the ground involved collophore anchoring in 8/15 sequences (Fig. 5C). Of the seven sequences without initial collophore anchoring, the collophore was used in the final arrest of four of those sequences, leaving three sequences without any evidence of collophore use. Sequences with initial collophore anchoring resulted in a shorter average landing distance 1.65 mm (min: 0.76 mm, max: 3.63 mm) than those without (mean: 17.62 mm, min: 6.2 mm, max: 27.75 mm). However, time to self-right after first contacting the ground spanned an overlapping range between the two landing strategies (initial collophore-anchoring mean: 0.072 s, min: 0.035 s, max: 0.162 s; no initial collophore anchoring: 0.127 s, min: 0.63 s, max: 0.24 s; Fig. 5C).

## Discussion

Jumping in globular springtails is achieved by rapidly unfolding the furca from underneath the body. For our study species, *D. minuta*, this accelerates their body to an average take-off velocity of 1 ms^−1^ in 1.7 ms. The fastest acceleration to liftoff we observed was 1938 ms^−2^. This ranks globular springtail acceleration among what many other fast-jumping arthropods experience: greater than insects such as fleas (Sutton and Burrows 2011) and leafhoppers (Burrows 2007), but less than others, like froghoppers (Burrows 2006) and pygmy mole crickets (Burrows and Picker 2010). These insects use elastic systems to move components of their hind legs to jump, as the energy requirements for their jumps exceed what is possible through direct muscle action. Our calculations strongly suggest a similar use of spring actuation within *D. minuta*. Musculature associated with the fourth abdominal segment likely is deforming abdominal basal rods and plates to build this energy for a jump (Oliveira and Smith 2024). However, our PD measurements are a very conservative estimate of the energy involved in globular springtail jumps. We assumed a much higher mass of the muscles that contribute power to the spring elements and our tracking is restricted to the movement of the center of mass of the body, which ignores the extreme body rotations seen in the springtail's ultrafast jump.

In the process of taking off, the furca rotates an average of 135° from underneath the body to extend behind, at an average angular velocity of 5,478.5 radians/s. Proceeding a jump, when folded underneath the body, the furca extends anteriorly beyond the center of mass. When pushing off the ground, the point of contact and interaction with the ground is largely through the mucro, which is the most distal segment of the furca (Oliveira and Smith 2024). These factors lead to furca extension that sends the globular springtail into the air spinning in a backward flip. We recorded an average body rotation of 282.2 Hz and a maximum of 368.7 Hz. Both this body rotation and the center of mass acceleration match closely to measures of the jump of the psyllid (jumping plant lice) *Cacopsylla peregrina*, which accelerates at an average of 1919 ms^−2^ while rotating at an average of 336 Hz (Burrows 2012). This psyllid species has an average body mass of 0.7 mg, which also closely matches that of the *D. minuta* in our study. The jumps of both globular springtails and psyllids result in the fastest whole-body rotations among arthropods; however, there are key differences between the jumps of these organisms. Psyllids power their jumps by rapidly rotating their hind legs anteriorly to their center of mass, sending them forward in the pitch plane. In addition, they are winged insects, able to extend their wings to dampen and stop their body rotation during the jump trajectory (Burrows 2012). Globular springtails, like as shown here for *D. mimuta*, do not dampen their rotation in the air. Instead, *D. minuta* is continually rotating backward in the pitch plane for an average of 20.5 rotations across their entire trajectory.

Our performance measures for *D. minuta* jumps are relatively similar to those for the two other globular springtails, belonging to different families, for which some jump performance measures have been quantified (Table 1). However, those species are reported to rotate faster (472 Hz and 416 vs. 282 Hz average) and reach higher trajectories (97 and 115 mm vs. 33 mm average) than *D. minuta*. Performance measures for more species of segmented springtails have been reported. Christian (1979) measured six species across four families, Sudo et al. (2015) provided measures for one unidentified semi-aquatic species, and Ortega-Jimenez et al. (2022) measured the semi-aquatic species *I. retardatus*. In comparison, globular springtails have higher take-off speeds, accelerate to take-off faster, and experience higher body rotational velocities.

Our study indicates that *D. minuta* is incapable of jumping directly forward off a uniformly flat surface. Even when we induced jumps with a posterior stimulus jumps in response were never straight ahead, directly away from the stimulus. We suspect that this is a result of the length of the furca relative to the body length, as well as how it flexes and interacts with the substrate. A previous study that focuses on the release of the furca of *D. minuta* (Oliviera and Smith 2024) showed that as the tips of the furca (the mucro) are pushing the springtail off the ground, the furca only flexes by 15.5° at the dens-manubrium joint. This means that the furca remains largely rigid during liftoff, continually applying force anterior to the center of mass of the body. The variation in trajectories we report in Fig. 4, we suspect, is due to changes in body orientation relative to the ground prior to furca release (Fig. 4C–D). Since a straight-ahead, forward space seems inaccessible through jumping, we suggest that *D. minuta* jumps are exclusively escape jumps and are likely not used for directional locomotion around their habitat.

Jumping in directional locomotory contexts is common with other collembolans. For instance, some Poduromorph collembolans in the family Hypogastruridae are known to collectively migrate in winter. Migrations involve directed, sun-oriented locomotion where the springtails both walk and jump in a forward-facing direction (Hågar 1995; Zettel et al. 2000). Forward-facing directional jumps seem, in fact, to be the normal condition for most segmented springtail orders where jumps have been studied (e.g., Christian 1979, Bauer and Christian 1987; Sudo et al. 2015). Even segmented species that are semi-aquatic and jump off the surface of the water, jump in a forward-facing direction (Sudo et al. 2013b, 2015; Ortega-Jimenez et al. 2022). In fact, water-surface jumps seem to be one scenario where globular springtails can jump forward, as *Sminthurides aquaticus* has been documented performing only forward-facing jumps off water surfaces (Ant Lab 2021). We suspect that with globular springtail water jumps the furca deforms the water surface resulting in the normal force pushing off the water surface at an angle, as described in Ortega-Jimenez et al. (2022).

In this study, we describe two landing strategies used by *D. minuta*: uncontrolled and collophore-anchored. Strategies for controlling landings through surface adhesion have been documented in the Entomobryomorpha and Poduromorpha collembolan orders. In Pododuromorpha, *Ceratophysella sigillata* was observed using both controlled and uncontrolled tumbling landings (Zettel et al. 2000). Uncontrolled landings followed rapid escape jumps, while controlled landings were used in prepared, directional jumps. Landings were controlled with everted antennal and anal sticky vesicles that stuck the collembolan to a wall or the substrate upon the descent of a jump. A different strategy was recently described in Entomobryomorphs with the semiaquatic springtail *I. retardatus* (Ortega-Jimenez et al. 2022). This species uses its collophore to collect a droplet of water when jumping off water surfaces. This droplet allows them to direct their aerial descent as well as to adhere to the water surface upon landing.

For globular springtails in the order Symphypleona specific strategies for landing are less well described. However, several studies of the clover springtail *S. viridis* collectively hint at a collophore-anchoring strategy like what we have described here for *D. minuta*. In *S. viridis*, like *D. minuta*, the collophore consists of a forking pair of long, sticky, and eversible vesicles that can extend beyond the length of the body (Chen et al. 2019). It is used for self-grooming and water balance, and to rapidly self-right after being stuck on their backs (Brackenbury 1990). Christian (1979), when originally describing their jumping behavior, noted that *S. viridis* everted the collophore during their jump. He hypothesized that an everted and sticky collophore might be used while landing amongst vegetation. Later, Chen et al. (2019) intercepted *S. viridis* jumps mid-air with clear plastic film and noted that everted vesicles of the collophore adhered to the smooth vertical surface, stopping the jump, and stabilizing the springtail to the surface. Though observations and reports detailing ground landing strategies of *S. viridis* have not been made, it seems likely that this globular springtail also uses a collophore-anchoring strategy. As our data show (Fig. 5), collophore-anchoring allows for an immediate arrest and recovery to a standing position, in comparison to an uncontrolled landing where the springtail might tumble over 5 times longer in both distance and time to recover to a standing position. A more detailed investigation into the adhesive forces applied through the collophore to rapidly arrest tumbling after a jump would be an interesting avenue of research.

Given the extraordinary performance measures, aerial behavior, and landing strategies we report here for *D. minuta*, we hope to inspire similar attention to the study of other globular springtails. The descriptions and measures in this study are in uniform hard-surfaced lab environment, replicating the conditions used to initially describe many other arthropod jumps. Of course, jumping in natural environments involves much more variable conditions, which could reveal different behaviors or performance measures. Additional descriptive studies of both terrestrial and aquatic jumping collembolans, in their environments, will provide a basis for comparative analysis and a better understanding of how these incredible animal movement systems evolve.

## Supplementary Material

obae029_Supplemental_Files

## Data Availability

The data underlying this article are available in the article and in its online supplementary material.
