# Supplementary material for: Jumping Performance and Behavior of the Globular Springtail Dicyrtomina minuta
Source: Integr Org Biol. 2024 Aug 29;6(1):obae029. doi: 10.1093/iob/obae029 (PMC11360184; doi:10.1093/iob/obae029)
Supplement: obae029_Supplemental_Files [file obae029_supplemental_files.zip › Supplementary Figure 1.docx]

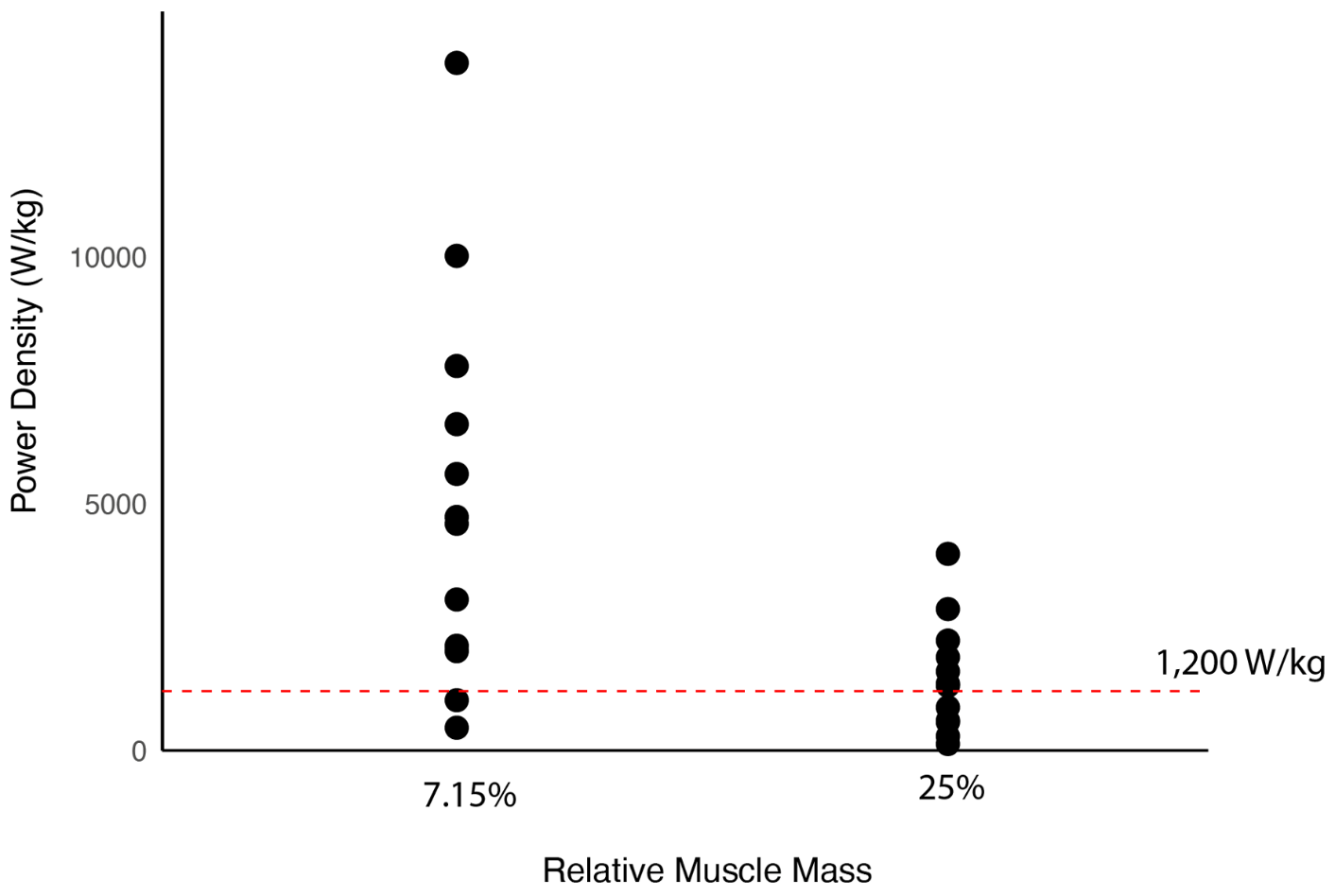


**Supplementary Figure 1:** Mass-specific power of *D. minuta* jumps assuming 7.15% and 25% of relative body mass is muscle powering the jump. With both of these conservative estimates of muscle mass, most power density measurements exceed the maximum measured power output recorded for vertebrate muscle (1200 W kg^-1^, red line on figure.).
