# Supplementary material for: Jumping Performance and Behavior of the Globular Springtail Dicyrtomina minuta
Source: Integr Org Biol. 2024 Aug 29;6(1):obae029. doi: 10.1093/iob/obae029 (PMC11360184; doi:10.1093/iob/obae029)
Supplement: obae029_Supplemental_Files [file obae029_supplemental_files.zip › RAW DATA + R code.zip!/FullJump/1-30-20_22.pdf]

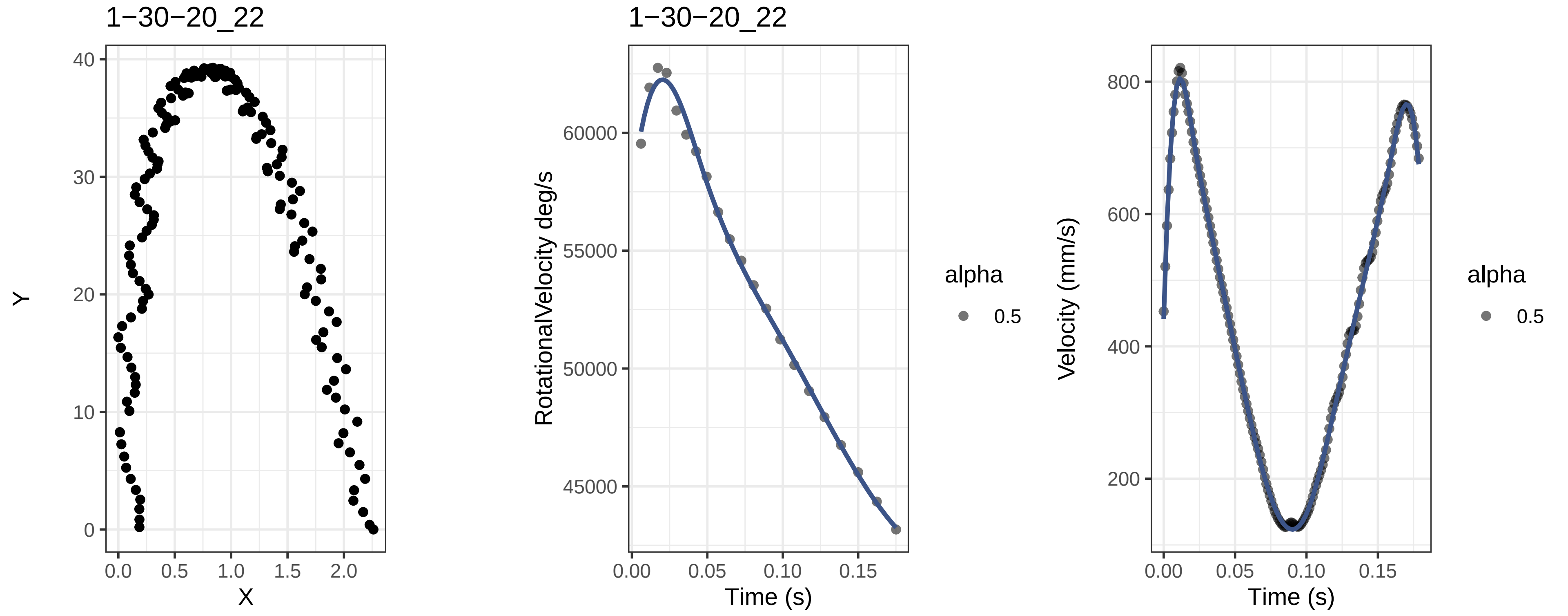
1−30−20_22
Y
X
1−30−20_22
RotationalVelocity deg/s
Time (s)
alpha
0.5
Velocity (mm/s)
Time (s)
alpha
0.5
