# Supplementary material for: Jumping Performance and Behavior of the Globular Springtail Dicyrtomina minuta
Source: Integr Org Biol. 2024 Aug 29;6(1):obae029. doi: 10.1093/iob/obae029 (PMC11360184; doi:10.1093/iob/obae029)
Supplement: obae029_Supplemental_Files [file obae029_supplemental_files.zip › RAW DATA + R code.zip!/FullJump/2-07-20_44.pdf]

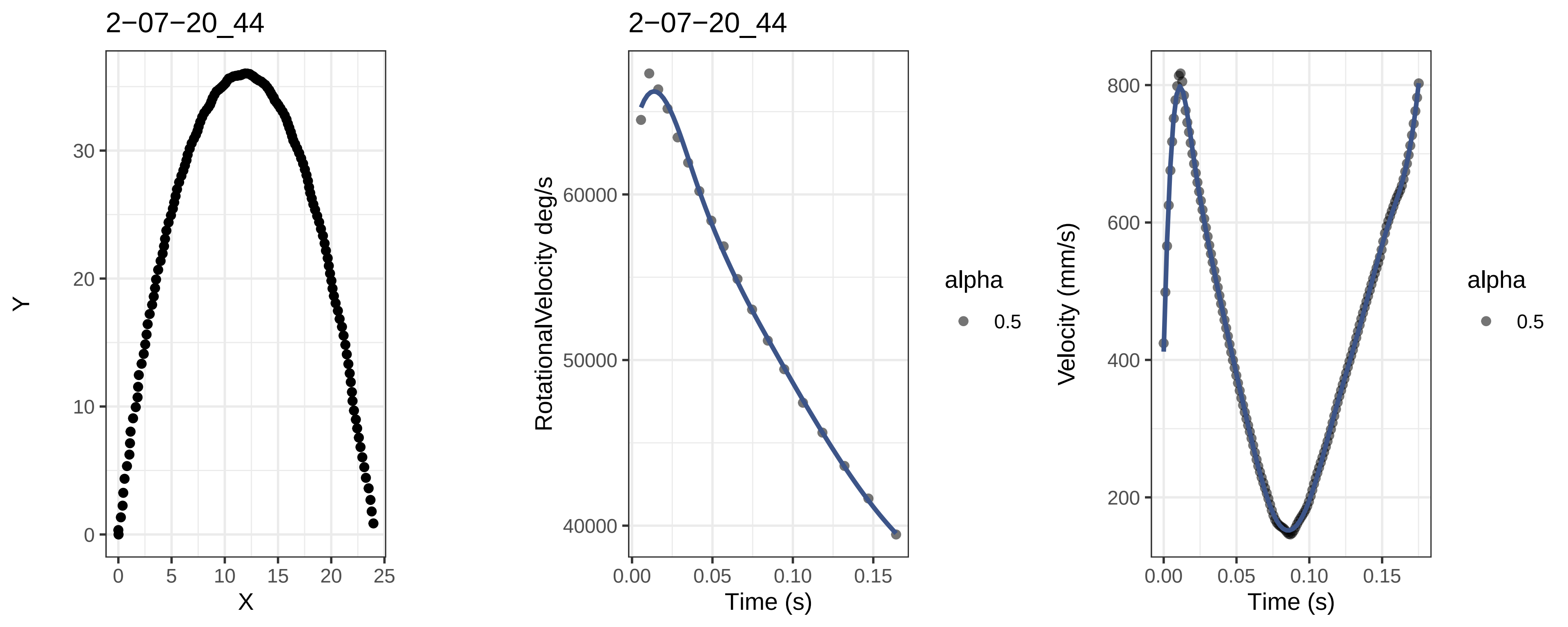
2−07−20_44
Y
X
0
10
20
30
0
5
10
15
20
25
2−07−20_44
RotationalVelocity deg/s
40000
50000
60000
0.00
0.05
0.10
0.15
Time (s)
alpha
0.5
Velocity (mm/s)
200
400
600
800
0.00
0.05
0.10
0.15
Time (s)
alpha
0.5
