# Supplementary material for: Jumping Performance and Behavior of the Globular Springtail Dicyrtomina minuta
Source: Integr Org Biol. 2024 Aug 29;6(1):obae029. doi: 10.1093/iob/obae029 (PMC11360184; doi:10.1093/iob/obae029)
Supplement: obae029_Supplemental_Files [file obae029_supplemental_files.zip › RAW DATA + R code.zip!/FullJump/2-07-20_47.pdf]

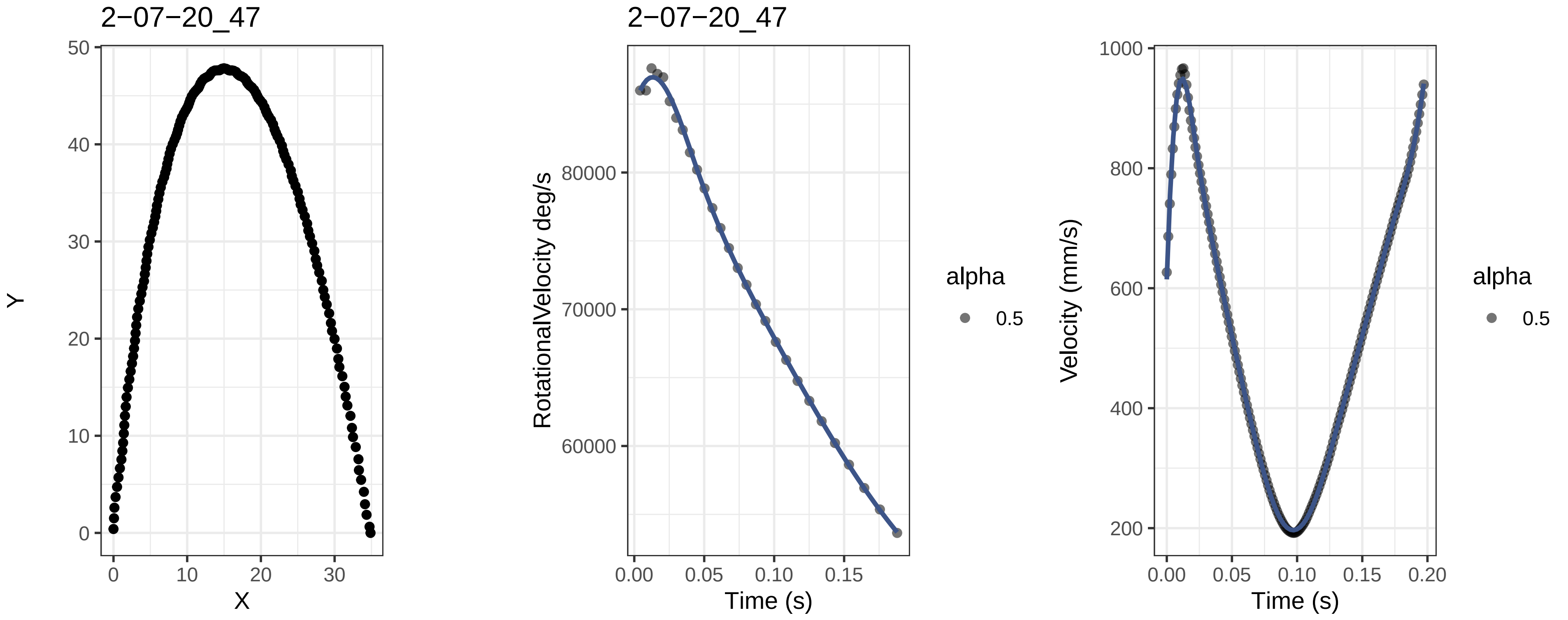

2−07−20_47
Y
X
50
40
30
20
10
0
0
10
20
30
2−07−20_47
RotationalVelocity deg/s
80000
70000
60000
Time (s)
0.00
0.05
0.10
0.15
alpha
0.5
Velocity (mm/s)
1000
800
600
400
200
Time (s)
0.00
0.05
0.10
0.15
0.20
alpha
0.5
