# Supplementary material for: Jumping Performance and Behavior of the Globular Springtail Dicyrtomina minuta
Source: Integr Org Biol. 2024 Aug 29;6(1):obae029. doi: 10.1093/iob/obae029 (PMC11360184; doi:10.1093/iob/obae029)
Supplement: obae029_Supplemental_Files [file obae029_supplemental_files.zip › RAW DATA + R code.zip!/TakeOff/ErrorAnalysis/ErrorTrack5_2-2-21_38_figure1.pdf]

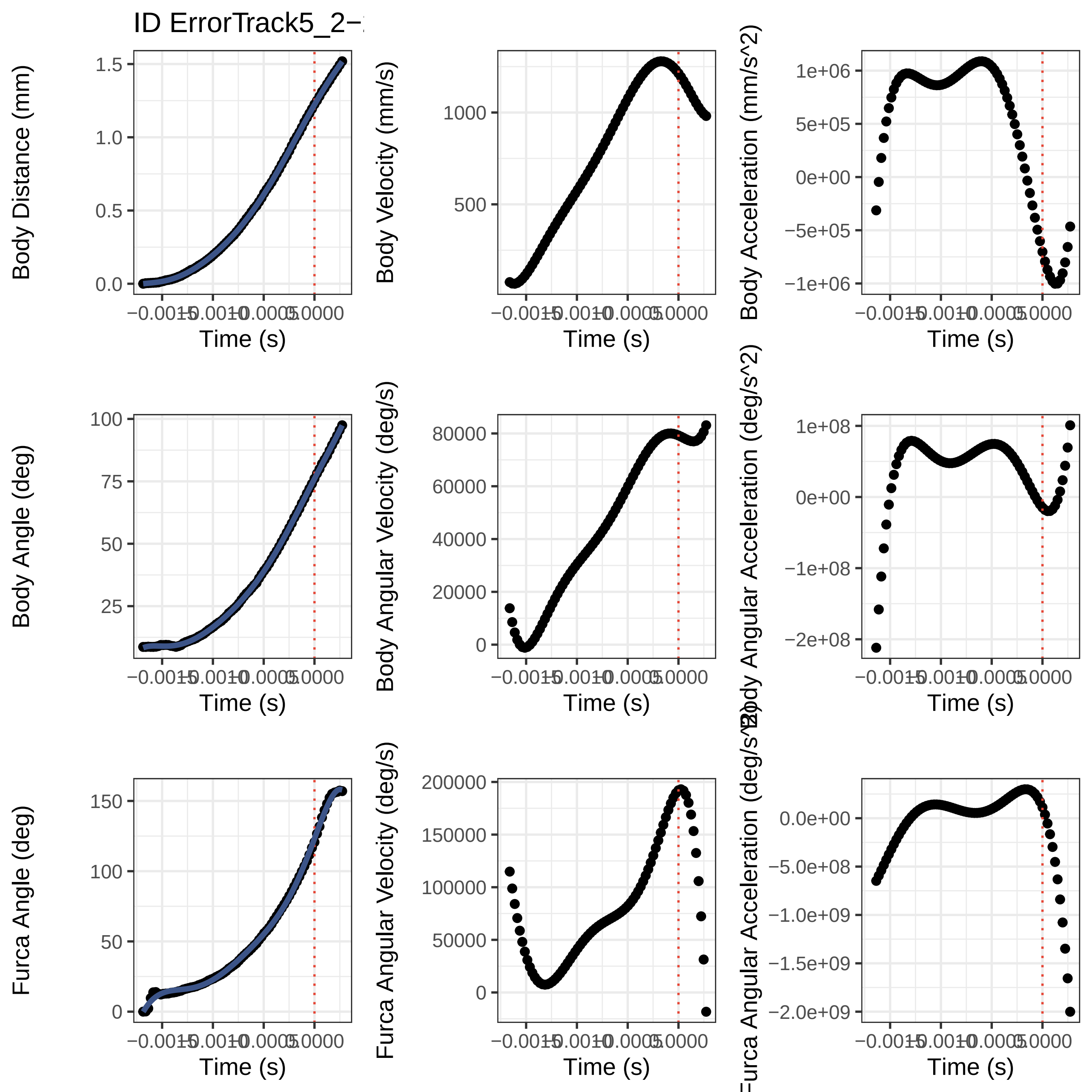

ID ErrorTrack5_2−
Body Distance (mm)
1.5
1.0
0.5
0.0
Body Velocity (mm/s)
1000
500
Body Acceleration (mm/s^2)
1e+06
5e+05
0e+00
−5e+05
−1e+06
Body Angle (deg)
100
75
50
25
Body Angular Velocity (deg/s)
80000
60000
40000
20000
0
Body Angular Acceleration (deg/s^2)
1e+08
0e+00
−1e+08
−2e+08
Furca Angle (deg)
150
100
50
0
Furca Angular Velocity (deg/s)
200000
150000
100000
50000
0
Furca Angular Acceleration (deg/s^2)
0.0e+00
−5.0e+08
−1.0e+09
−1.5e+09
−2.0e+09
−0.0015
−0.0010
−0.0005
0.0000
Time (s)
