# Supplementary material for: Jumping Performance and Behavior of the Globular Springtail Dicyrtomina minuta
Source: Integr Org Biol. 2024 Aug 29;6(1):obae029. doi: 10.1093/iob/obae029 (PMC11360184; doi:10.1093/iob/obae029)
Supplement: obae029_Supplemental_Files [file obae029_supplemental_files.zip › RAW DATA + R code.zip!/TakeOff/Tracks/2-2-21_37_figure1.pdf]

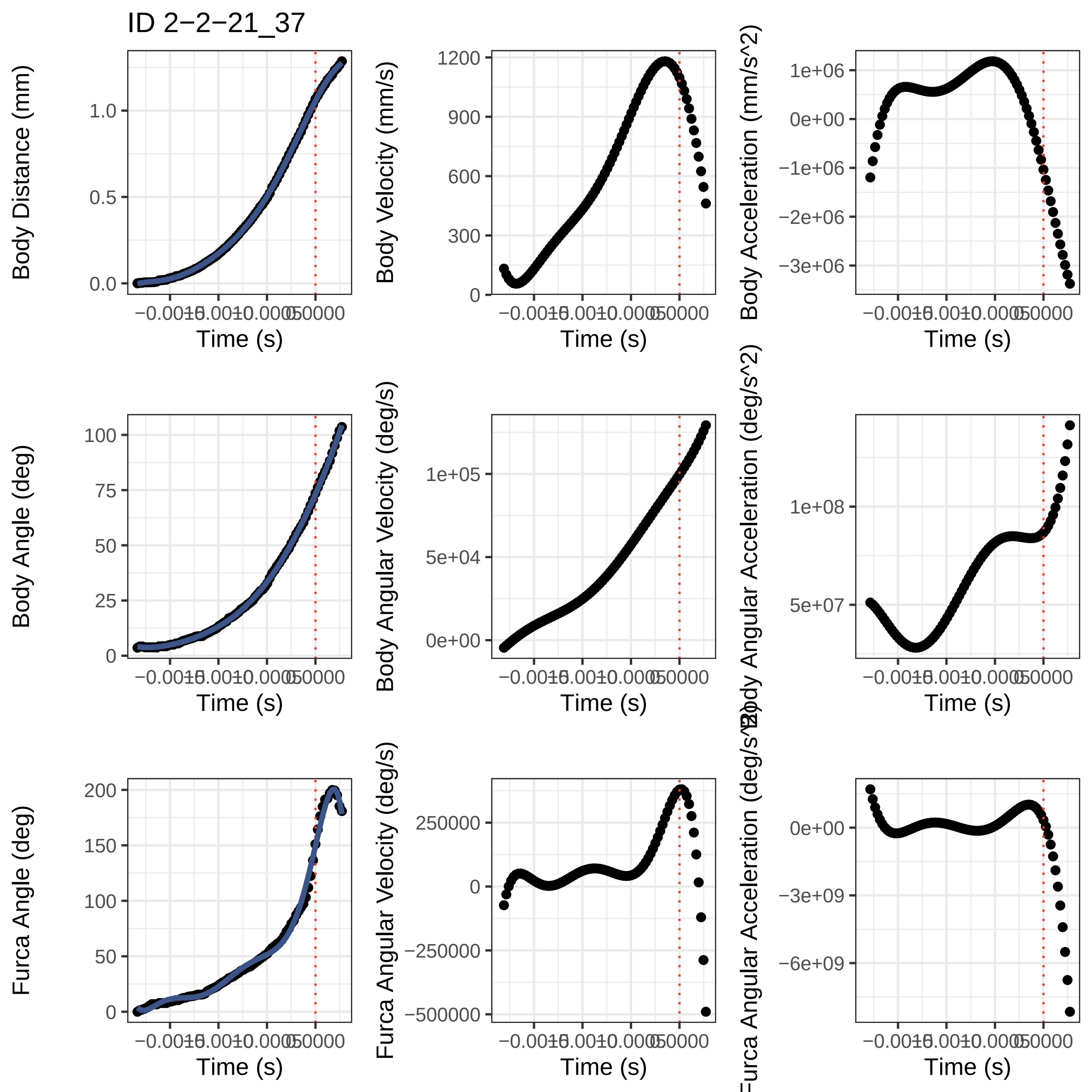
ID 2−2−21_37
Body Distance (mm)
Body Velocity (mm/s)
Body Acceleration (mm/s^2)
Body Angle (deg)
Body Angular Velocity (deg/s)
Body Angular Acceleration (deg/s^2)
Furca Angle (deg)
Furca Angular Velocity (deg/s)
Furca Angular Acceleration (deg/s^2)
Time (s)
0.0
0.5
1.0
0
300
600
900
1200
1e+06
0e+00
−1e+06
−2e+06
−3e+06
0
25
50
75
100
0e+00
5e+04
1e+05
5e+07
1e+08
0
50
100
150
200
250000
0
−250000
−500000
0e+00
−3e+09
−6e+09
