# Supplementary material for: Jumping Performance and Behavior of the Globular Springtail Dicyrtomina minuta
Source: Integr Org Biol. 2024 Aug 29;6(1):obae029. doi: 10.1093/iob/obae029 (PMC11360184; doi:10.1093/iob/obae029)
Supplement: obae029_Supplemental_Files [file obae029_supplemental_files.zip › RAW DATA + R code.zip!/TakeOff/Tracks/2-2-21_43_figure1.pdf]

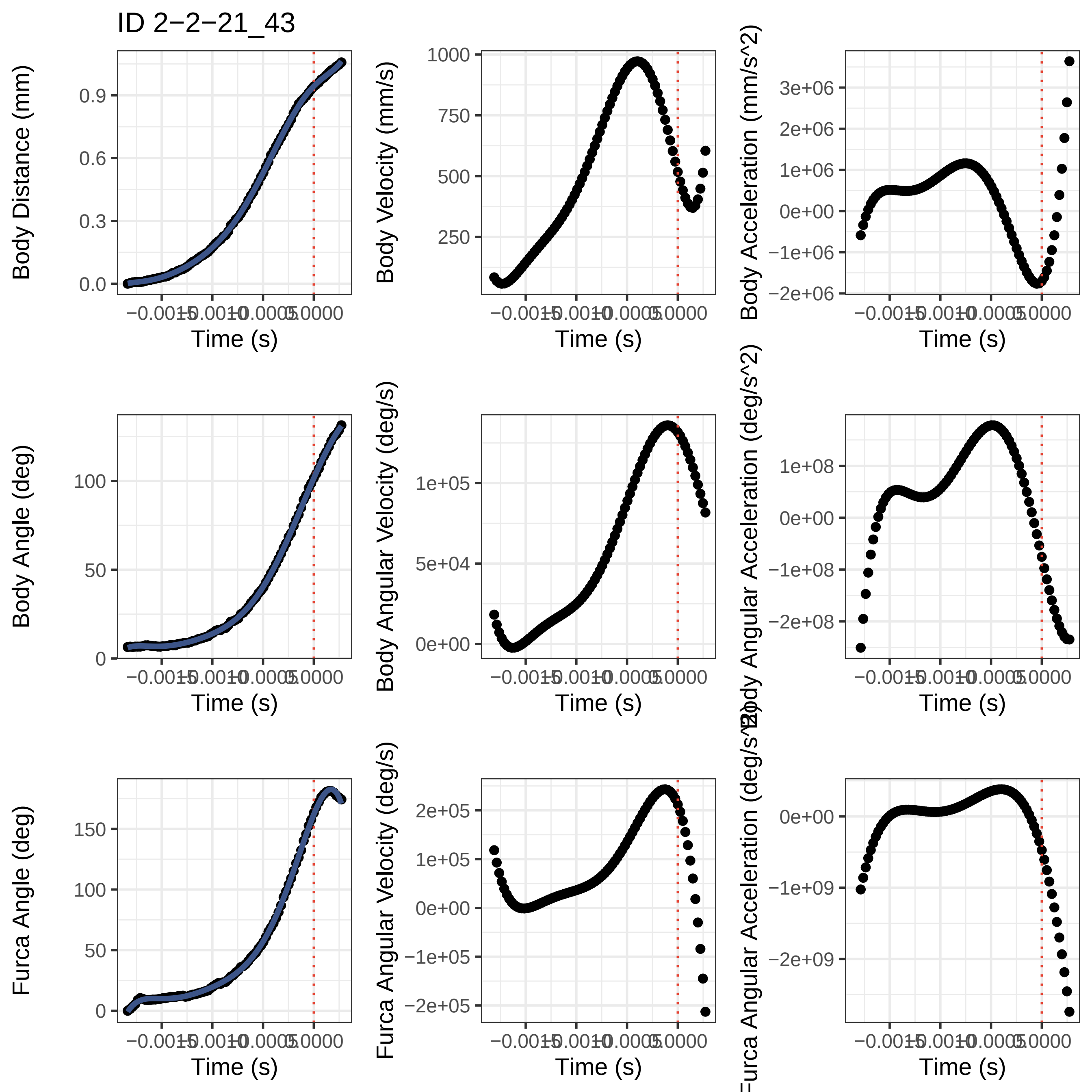
ID 2−2−21_43
Body Distance (mm)
Body Velocity (mm/s)
Body Acceleration (mm/s^2)
Body Angle (deg)
Body Angular Velocity (deg/s)
Body Angular Acceleration (deg/s^2)
Furca Angle (deg)
Furca Angular Velocity (deg/s)
Furca Angular Acceleration (deg/s^2)
Time (s)
−0.0015
−0.0010
−0.0005
0.0000
0.0
0.3
0.6
0.9
250
500
750
1000
−2e+06
−1e+06
0e+00
1e+06
2e+06
3e+06
0
50
100
0e+00
5e+04
1e+05
−2e+08
−1e+08
0e+00
1e+08
150
−2e+05
−1e+05
1e+05
2e+05
−2e+09
−1e+09
