# Supplementary material for: Jumping Performance and Behavior of the Globular Springtail Dicyrtomina minuta
Source: Integr Org Biol. 2024 Aug 29;6(1):obae029. doi: 10.1093/iob/obae029 (PMC11360184; doi:10.1093/iob/obae029)
Supplement: obae029_Supplemental_Files [file obae029_supplemental_files.zip › RAW DATA + R code.zip!/TakeOff/Tracks/2-3-21_49_figure1.pdf]

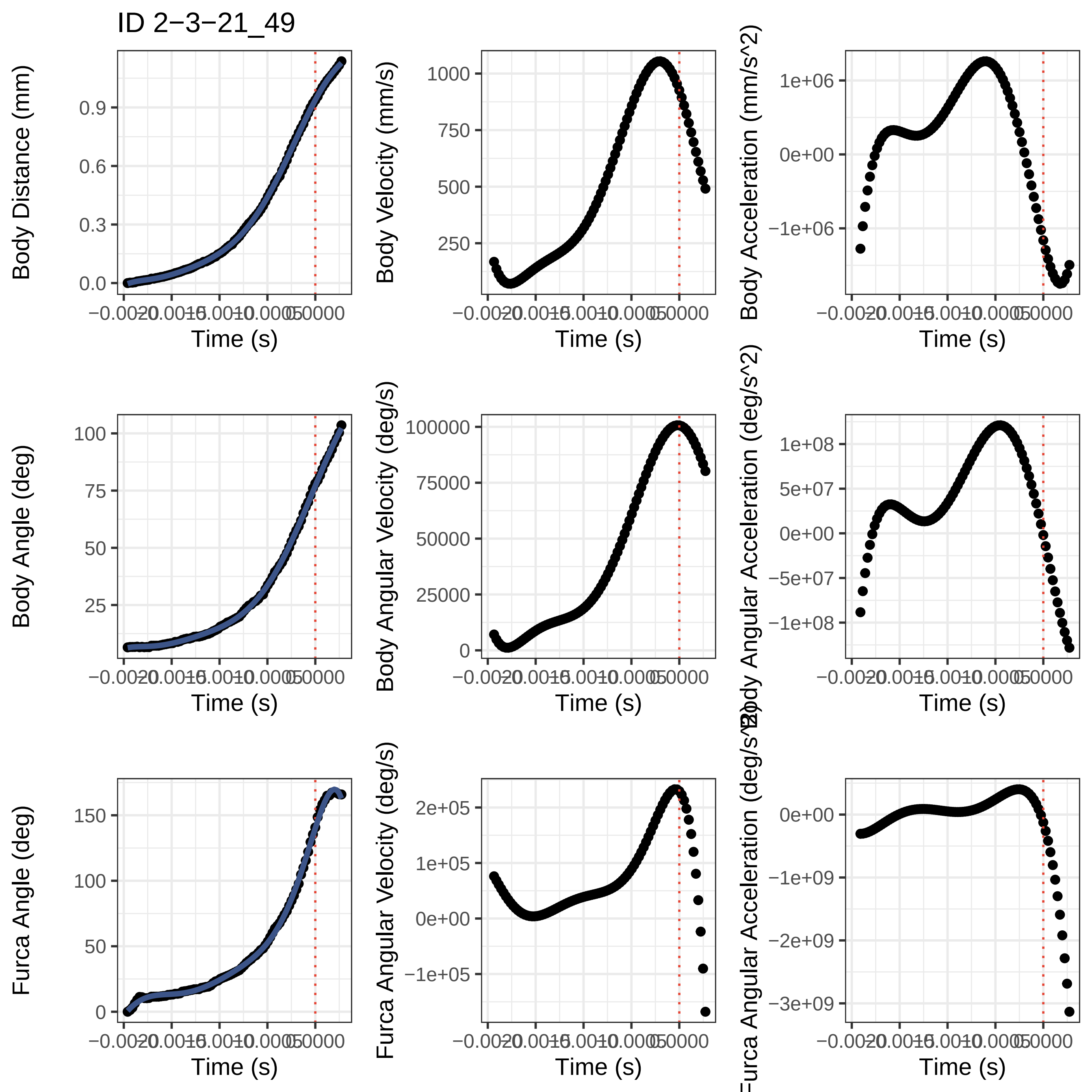

ID 2−3−21_49
Body Distance (mm)
Body Velocity (mm/s)
Body Acceleration (mm/s^2)
Body Angle (deg)
Body Angular Velocity (deg/s)
Body Angular Acceleration (deg/s^2)
Furca Angle (deg)
Furca Angular Velocity (deg/s)
Furca Angular Acceleration (deg/s^2)
Time (s)
