# Supplementary material for: Jumping Performance and Behavior of the Globular Springtail Dicyrtomina minuta
Source: Integr Org Biol. 2024 Aug 29;6(1):obae029. doi: 10.1093/iob/obae029 (PMC11360184; doi:10.1093/iob/obae029)
Supplement: obae029_Supplemental_Files [file obae029_supplemental_files.zip › RAW DATA + R code.zip!/TakeOff/Tracks/2-3-21_51_figure1.pdf]

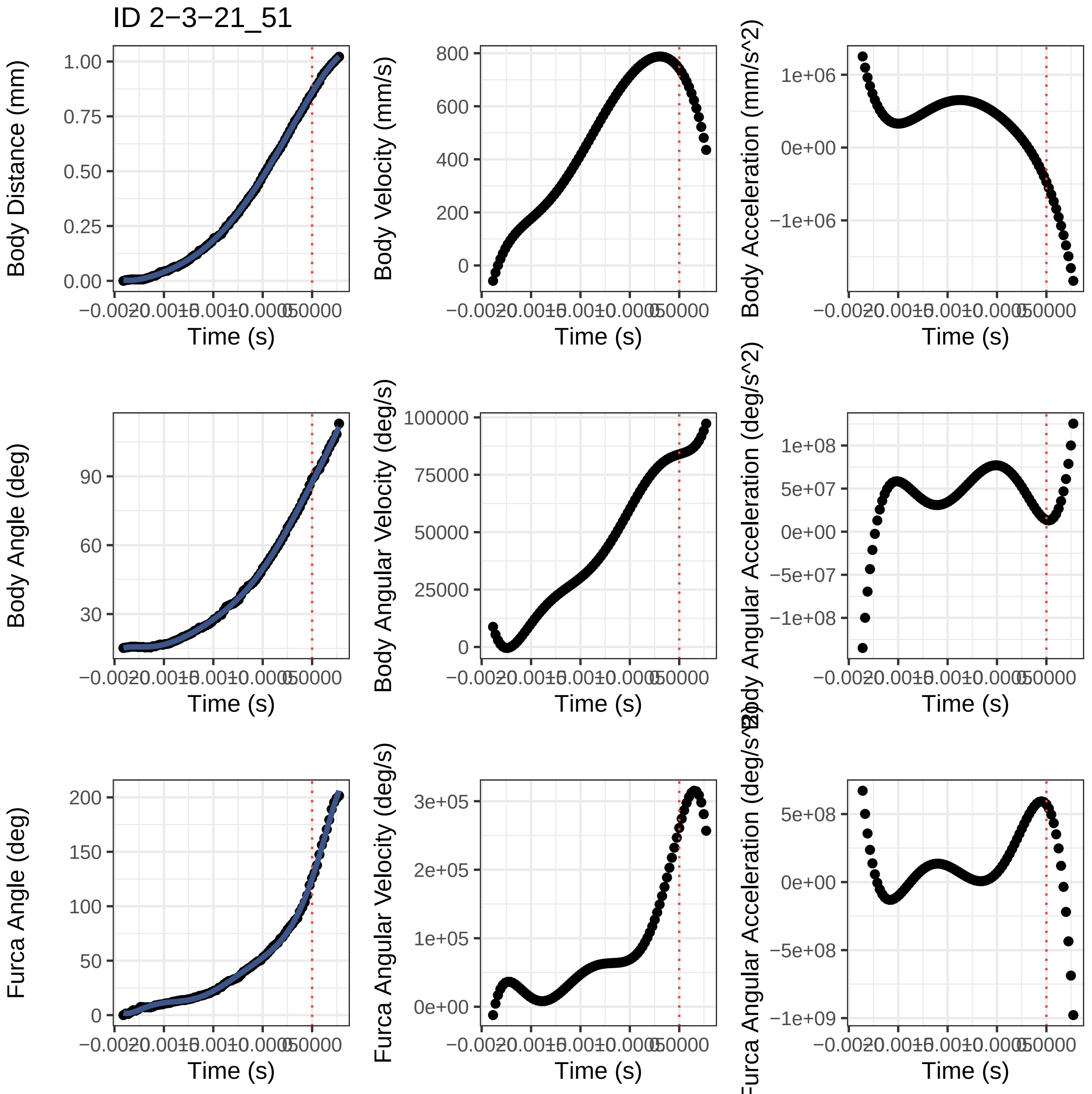
ID 2−3−21_51
Body Distance (mm)
1.00
0.75
0.50
0.25
0.00
Body Velocity (mm/s)
800
600
400
200
0
Body Acceleration (mm/s^2)
1e+06
0e+00
−1e+06
Body Angle (deg)
90
60
30
Body Angular Velocity (deg/s)
100000
75000
50000
25000
0
Body Angular Acceleration (deg/s^2)
1e+08
5e+07
0e+00
−5e+07
−1e+08
Furca Angle (deg)
200
150
100
50
0
Furca Angular Velocity (deg/s)
3e+05
2e+05
1e+05
0e+00
Furca Angular Acceleration (deg/s^2)
5e+08
0e+00
−5e+08
−1e+09
Time (s)
