# Supplementary material for: Jumping Performance and Behavior of the Globular Springtail Dicyrtomina minuta
Source: Integr Org Biol. 2024 Aug 29;6(1):obae029. doi: 10.1093/iob/obae029 (PMC11360184; doi:10.1093/iob/obae029)
Supplement: obae029_Supplemental_Files [file obae029_supplemental_files.zip › RAW DATA + R code.zip!/TakeOff/Tracks/2-3-21_52_figure1.pdf]

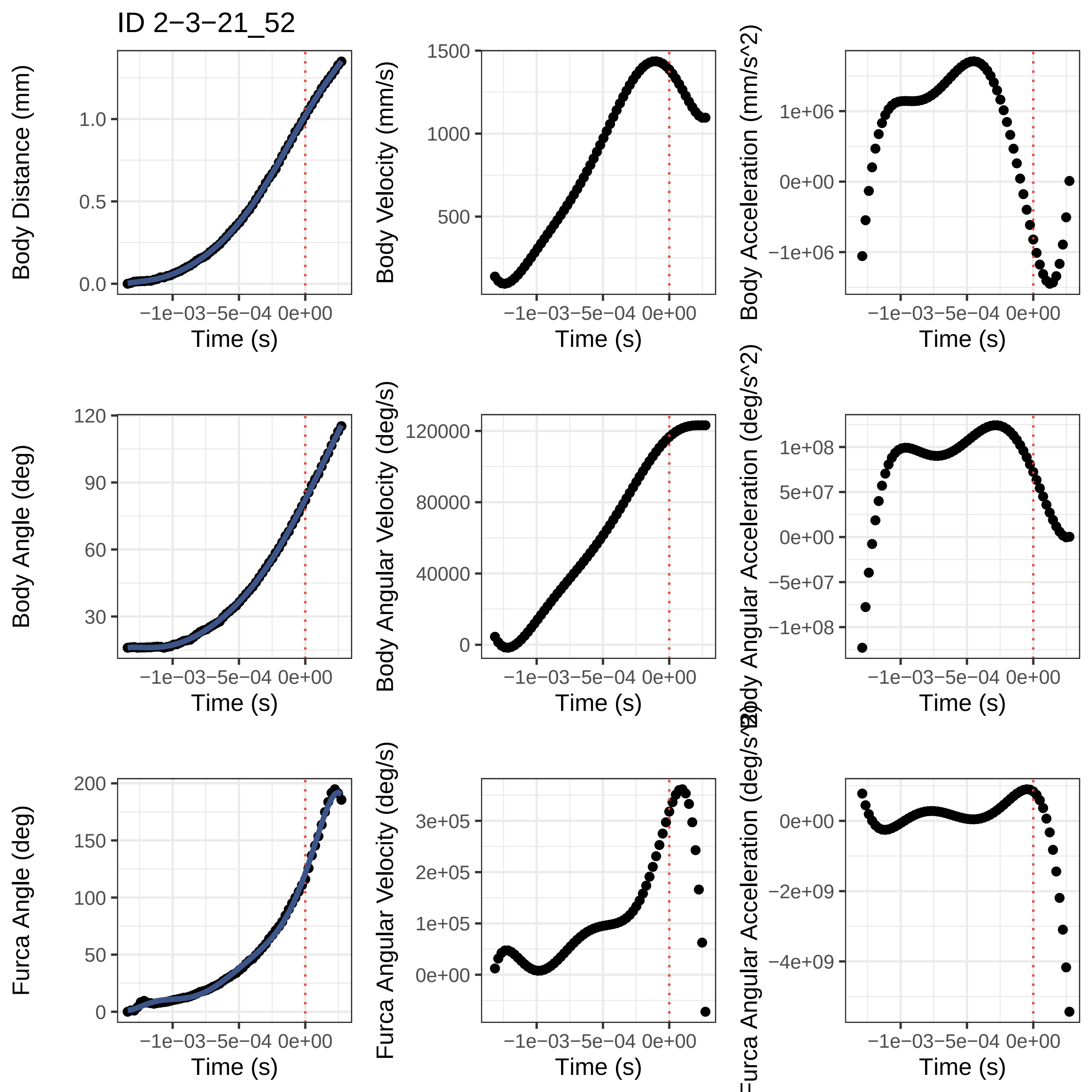
ID 2−3−21_52
Body Distance (mm)
Body Velocity (mm/s)
Body Acceleration (mm/s^2)
Body Angle (deg)
Body Angular Velocity (deg/s)
Body Angular Acceleration (deg/s^2)
Furca Angle (deg)
Furca Angular Velocity (deg/s)
Furca Angular Acceleration (deg/s^2)
Time (s)
−1e−03 −5e−04 0e+00
0.0 0.5 1.0
500 1000 1500
−1e+06 0e+00 1e+06
30 60 90 120
0 40000 80000 120000
−1e+08 −5e+07 0e+00 5e+07 1e+08
0 50 100 150 200
0e+00 1e+05 2e+05 3e+05
−4e+09 −2e+09 0e+00
